# Supplementary material for: Dietary Taurine Intake Affects the Growth Performance, Lipid Composition, and Antioxidant Defense of Juvenile Ivory Shell (Babylonia areolata)
Source: Animals (Basel). 2023 Aug 11;13(16):2592. doi: 10.3390/ani13162592 (PMC10451277; doi:10.3390/ani13162592)
Supplement: Supplementary file 1 [file animals-13-02592-s001.zip › animals-2540828-supplementary.pdf]

Table S1: Ingredients and composition of experimental diets.

Ingredients (%)		Diets
Forage fish meat (Decapterus maruadsi)		58.00
Soy protein concentrate		12.00
α-starch		10.00
Krill meal		4.00
Squid splanchnic powder		3.50
Monocalcium phosphate		2.00
Spirulina		2.00
Vitamin premix		2.00
Fish oil		4.00
Gelatine		2.50
Total		100
Moisture (%)		52.47±0.04
Crude protein (%)	WW	24.84±0.21
	DW	52.26±0.40
Crude lipid (%)	WW	3.52±0.04
	DW	7.41±0.08
Ash (%)	WW	15.95±0.33
	DW	33.56±0.67
Taurine (mg/g)	WW	0.61±0.06
	DW	1.28±0.12

WW, % wet weight; DW, % dry weight.

Table S2: Fatty acid profiles (%) of soft artificial feed without taurine supplement

**Fatty acids**	**% of total fatty acids**
C14:0	2.05±0.07
C15:0	0.36±0.03
C16:0	29.68±0.63
C17:0	0.39±0.02
C18:0	3.59±0.04
SFA	36.06±0.72
C16:1n7	2.76±0.10
C17:1n7	0.13±0.00
C18:1n9 (z)	9.72±0.41
C18:1n9 (e)	2.73±0.12
C20:1n9	1.00±0.06
MUFA	16.34±0.62
C16:3n3	0.40±0.01
C18:2n6	16.50±0.09
C18:3n6	3.29±0.03
C18:3n3	1.38±0.05
C20:2n6	0.20±0.00
C20:4n6	1.82±0.03
C20:5n3	4.12±0.06
C22:5n-3	2.93±0.04
C22:6n-3	16.95±0.22
PUFA	47.59±0.49

Table S3: Sequences of primers used for real-time quantitative PCR.

Gene	Sequence (5′- 3′)	Product size (bp)
Leptin	F: GAAACGACGATGCCTGTGG<br>R: TGTCCTGTTCTTCTACGTGCTG	129
Orexin	F: CAGATGCGATAATAGCGGTCA<br>R: AATTGCCGATCTGCGCTT	138

NPY	F: TGCCGAAGACCCAGGAGTT<br>R: TGGTGGTGATCGTGGTGCT	150
Cholecystokinin	F: ATGACCTTGACCTCCGTGG<br>R: AACCTCCACCACCGCTTTC	123
β-actin	F: TCTTGGGTATGGAATCTGCTGG<br>R: CCTTTTGCATTCTGTCAGCGAT	130

NPY: *Neuropeptide Y*.
